# Embedding equity and inclusion in universities through motivational theory and community‐based conservation approaches

**DOI:** 10.1111/cobi.14384

**Published:** 2024-11-25

**Authors:** Maï Yasué, Netta Weinstein, Sara E. Harris, I‐Chant A. Chiang, Nicole Legate, Ashley J. Moore, Nadia Joe

**Affiliations:** ^1^ Faculty of Medicine The University of British Columbia Vancouver British Columbia Canada; ^2^ Psychology University of Reading Reading UK; ^3^ Faculty of Science The University of British Columbia Vancouver British Columbia Canada; ^4^ Sprott Shaw College Burnaby British Columbia Canada; ^5^ Equity & Inclusion Office The University of British Columbia Vancouver British Columbia Canada; ^6^ Psychology, Illinois Institute of Technology Chicago USA; ^7^ Yukon University Whitehorse Yukon Canada

**Keywords:** DEI, EDI, human rights, ICDP, justice, DEI, derechos humanos, EDI, ICDP, justicia, ICDP, DEI, EDI, 人权, 公正

## Abstract

Despite widespread plans to embed justice, equity, decolonization, indigenization, and inclusion (JEDII) into universities, progress toward deeper, systemic change is slow. Given that many community‐based conservation (CBC) scholars have experience creating enduring social change in diverse communities, they have transferable skills that could help embed JEDII in universities. We synthesized the literature from CBC and examined it through the lens of self‐determination theory to help identify generalizable approaches to create resilient sociocultural change toward JEDII in universities. Fostering autonomous motivation (i.e., behaving because one truly values and identifies with the behavior or finds behavior inherently satisfying) is critical to inspiring enduring change in both CBC and JEDII. Based on theory and our examination of CBC, we provide 5 broad recommendations that helped motivate behavioral change in a way that was self‐sustained (i.e., even without external rewards or pressure). Guiding principles support autonomy by creating meaningful choice and different entry points for JEDII; prioritising relationships; designing payment programs that enhance autonomous motivation; developing meaningful educational opportunities that are relevant, timely, relational, and authentic; and creating institutional change by focusing efforts on critical moments.

## Abstract (es)

Integración de la equidad y la inclusión en las universidades por medio de la teoría motivacional y las estrategias de conservación comunitaria

A pesar de los planes generalizados para integrar la justicia, la equidad, la descolonización, la indigenización y la inclusión (JEDII) en las universidades, el progreso hacia un cambio sistémico más profundo es lento. Muchos académicos de la conservación basada en comunidades (CBC) tienen experiencia en la creación de un cambio social duradero en diversas comunidades, por lo que poseen habilidades transferibles que podrían ayudar a integrar la JEDII en las universidades. Sintetizamos la bibliografía de la CBC y la analizamos a través de la lente de la teoría de la autodeterminación para ayudar a identificar enfoques generalizables para crear un cambio sociocultural resistente hacia la JEDII en las universidades. El fomento a la motivación autónoma (es decir, comportarse porque uno realmente valora y se identifica con el comportamiento o encuentra el comportamiento inherentemente satisfactorio) es fundamental para inspirar un cambio duradero tanto en la CBC como en el JEDII. Con base en la teoría y en nuestro examen de la CBC, ofrecemos cinco recomendaciones generales que ayudan a motivar el cambio de comportamiento de forma autosostenida (es decir, incluso sin recompensas o presiones externas). Los principios rectores son apoyar la autonomía con la creación de opciones significativas y diferentes puntos de entrada para JEDII; dar prioridad a las relaciones; diseñar programas de pago que potencien la motivación autónoma; desarrollar oportunidades educativas significativas que sean relevantes, oportunas, relacionales y auténticas; y crear un cambio institucional con los esfuerzos enfocados en momentos críticos.

## 利用激励理论和基于社区的保护方法在大学中实现公平和包容

【摘要】 尽管将公正、公平、非殖民化、本土化和包容(justice, equity, decolonization, indigenization and inclusion, JEDII)纳入大学的计划已非常普遍, 但在实现更深层次的系统性变革方面仍进展缓慢。鉴于许多基于社区的保护(community‐based conservation, CBC)学者拥有在不同社区创造持续社会变革的经验, 他们的技能也可以用于帮助大学更好地推动JEDII。我们综述了CBC方面的文献, 并利用自我决定论的视角对其进行研究, 以帮助确定可以在大学中创造针对JEDII的弹性社会文化变革的通用方法。培养自主动机(即因真正重视和认同某种行为或认为该行为能带来内在满足而采取行动)对于激发CBC和JEDII的持续变革至关重要。基于理论和我们对CBC的研究, 我们提出了五项广泛的建议, 来帮助激励自我维持(不需要外部奖励或压力)的行为改变。这些指导原则包括:通过为JEDII创造有意义的选择和不同切入点来支持自主性;优先考虑人际关系;设计能够提高自主性动机的支付计划;开发适宜、及时、相关和真实的有意义的教育机会;通过把握关键时刻来创造制度性变革。**【翻译:胡怡思;审校:聂永刚】**

## INTRODUCTION

Universities across North America and Europe are developing a myriad of strategies and action plans to spark change toward JEDII (justice, equity, decolonization, Indigenization, and inclusion). Yet, actual implementation of these institutional and interpersonal changes remains slow. Community‐based conservation (CBC) scholars can promote antioppressive, equitable, and decolonized practices that support self‐determination and inclusion of historically, persistently, and systemically marginalized groups (hereafter marginalized groups) in their global research and teaching (Cronin et al., 2021; Crosman et al., 2022; Ignace et al., 2023; Johri et al., 2021). To go beyond research and teaching, we focused on how CBC scholars can also play a role in advancing university‐wide, enduring cultural change to support JEDII.

We adopted the acronym *JEDII* as opposed to *EDI* (equity, diversity, inclusion) to differentiate between actions to advance decolonization and indigenization (Gaudry & Lorenz, 2018; Mulligan, 2002; Schneider, 2023) and broader inclusion work. We included *justice* to emphasize the importance of redressing historic and ongoing injustices in conservation (West et al., 2006). As others note, the field of conservation is complicit in colonization and racism in its support of the first protected areas and ongoing evictions of Indigenous, racialized, and impoverished people (Dowie, 2011; Mulligan, 2002; Smallhorn‐West et al., 2023).

Collectively, we are community‐based, environmental justice, conservation, and motivation scholars and JEDII strategists and leaders in postsecondary institutions in Canada, the United States, and the United Kingdom. All of us come from academic fields that are not directly related to JEDII; however, as members of marginalized groups, we became engaged in JEDII work at our institutions. For us, like other members of marginalized groups, our identities (Rodríguez et al., 2015) and experiences of marginalization catalyzed our engagement in JEDII work. Through this article, we sought to strengthen the connection between the fields of CBC and JEDII by synthesizing the literature from CBC within a powerful, accessible, and evidence‐based theoretical framework through which we identify broad recommendations that may help create more enduring JEDII initiatives. We hope to help other conservation scholars see connections between their work and JEDII and inspire more action.

## COMMUNITY‐BASED CONSERVATION

Partly in response to top‐down “fences and fines” conservation, CBC aims to work with local communities to create socioenvironmental change (Adams et al., 2004; Berkes, 2004; Brosius et al., 2005). CBC draws from fields including political science, ecology, economics, and psychology and increasingly from Indigenous governance to identify the best approaches to have material (e.g., payments, construction of a new school) or nonmaterial (e.g., education, ecotourism, self‐governance) gains for communities that also benefit the environment (Alcorn, 1993; DeCaro & Stokes, 2008; Kimmerer, 2013; Nielsen et al., 2021, p. 202; Ostrom, 1990; Trosper, 2003). Today, extensive research, including meta‐analyses and longitudinal studies (Brooks et al., 2013, 2012; Kellert et al., 2000; Reggers et al., 2018), describes design characteristics of effective and ineffective CBC projects (Brown, 2002; Kellert et al., 2000; Western & Wright, 2013).

## UNIVERSITY‐WIDE JEDII ACTION

In comparison, although there are studies demonstrating the mixed or limited impacts of JEDII interventions on short‐term attitudinal changes, there have been comparably few empirical, longitudinal studies on the effectiveness of postsecondary JEDII initiatives (Chang et al., 2019; Dobbin & Kalev, 2018; Legate & Weinstein, 2024). The few meta‐analyses that exist tend to focus on antibias training (Bezrukova et al., 2016; Carter et al., 2020), which is one small component of JEDII‐related change (Dobbin & Kalev, 2022; Stewart & Valian, 2022). The paucity of research findings is partly because universities have only started over the last 5 years to hire senior leaders in EDI with some power to embed changes in institutions, and very few have large enough EDI personnel to provide advice to support decision makers in creating change (al Shaibah, 2024).

There are key similarities between the context of CBC and advancing JEDII in universities that allowed us to bring examples from CBC to identify promising practices for JEDII (Chiorean et al., 2021; Kolluru et al., 2023). First, conservation practitioners work to create sociocultural change with diverse groups (e.g., resource users; environmental nongovernmental, colonial, and Indigenous governmental organizations; and private sectors) who have disparate and sometime conflicting goals, values, cultures, and histories (Brosius et al., 2005; Western & Wright, 2013; Wolbring & Nguyen, 2023) by designing processes that provide agency and voice to people with less recognized power (Austin et al., 2018; Reed et al., 2019). Similar to CBC, units across universities work to integrate JEDII in partnership with centralized support units (e.g., equity and inclusion offices), decentralized units, and less institutionalized structures, such as community groups or affinity groups (e.g., Black student's society) (Fadoju et al., 2021; Lingras et al., 2023), with unique cultures and histories. Second, decades of CBC research suggest that diverse perspectives and lived experiences are critical to advance conservation (Alcorn, 1993; Apostolopoulou et al., 2021; Schneider, 2023; Shepherd, 2004). Similarly, some universities have started to connect diversity (in all forms) to excellence in teaching, community engagement, and research (Hurtado, 2007; Reyes‐García & Benyei, 2019; Stewart & Valian, 2022). Third, both conservation and JEDII are applied, interdisciplinary academic fields that rely on quantitative and qualitative methods and lack a universal description of effectiveness (Brooks et al., 2013). Diverse disciplinary methodologies and concepts of supposed success can thus lead to perpetual task forces and continual data collection with limited action (Gwayi‐Chore et al., 2021; Mmeje et al., 2020). Lessons learned in CBC about the trade‐off between perfectionism, planning, and research versus imperfect action are especially relevant in advancing JEDII in universities. Delayed action and ongoing exclusion affect both academic excellence and the lives of marginalized groups (Gómez, 2023; Graham et al., 2023) in the same way that delayed action for planning and research in CBC can lead to rapid environmental degradation (Hansen et al., 2011; Possingham et al., 2007).

## THEORETICAL MOTIVATIONAL FRAMEWORK

**FIGURE 1:**
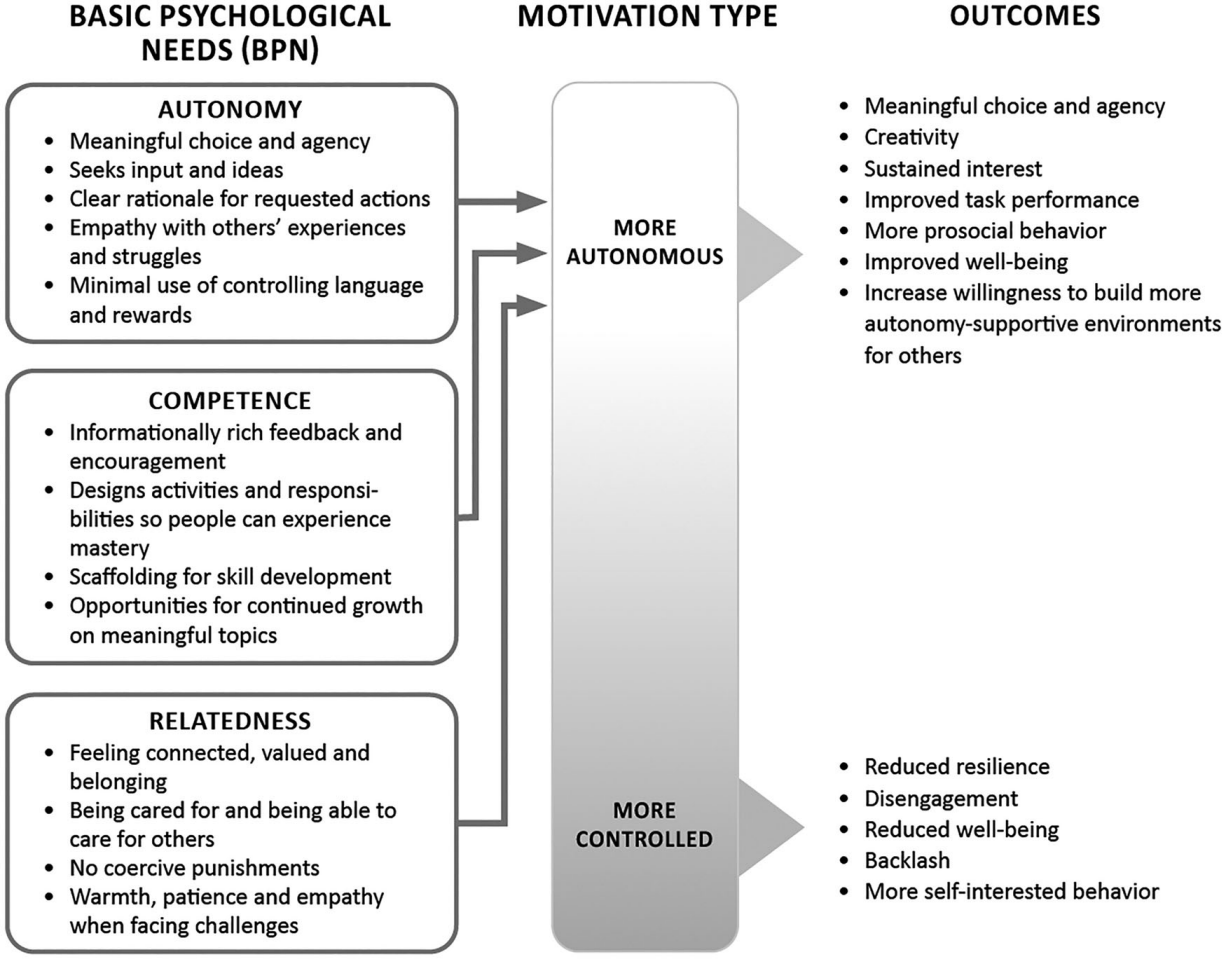
The relationship between the basic psychological needs of autonomy, competence, and relatedness; autonomous motivation; and outcomes.

Understanding how to motivate people is essential to CBC and JEDII because effecting change at individual and institutional levels requires sustained effort and commitment. Furthermore, although there are many examples and promising practices from CBC, a theoretical framework is necessary to help identify the approaches that are most likely to have generalizable benefits into the relatively novel context of JEDII. Self‐determination theory (Ryan & Deci, 2000) is a theory of motivation concerned with the social conditions that create enduring behavioral change. Hundreds of empirical studies (Ryan & Deci, 2017) in organizations, healthcare, environmental conservation, parenting, and education demonstrate that changes in behavior that happen for autonomous or well‐internalized reasons tend to be effective in the short and long term (Chamberlin et al., 2023; DeCaro & Stokes, 2008; Joussemet et al., 2008; Šakan et al., 2020; Van den Broeck et al., 2016). When people are autonomously motivated, they act because they understand and endorse the value of a behavior. This type of motivation can be contrasted with controlled forms of motivation, including acting because of rewards, pressure, judgment, or feeling guilt or shame in front of others (Deci, 1971; Deci et al., 2001). When the two are compared, autonomous motivation leads to greater performance and engagement, greater persistence to carry out tasks, more collaboration, higher organization or task performance, and greater commitment to organizations and personal growth, as well as higher prosocial and pro‐environmental behavior and improved psychological health and well‐being (Aitken et al., 2016, p. 20; Marshall et al., 2017; Moreau & Mageau, 2012; Rohe et al., 2006; Tannock, 2017; Vallerand et al., 1997). Although controlled forms of motivation can lead to behavioral changes (Elias, 2021; Rai et al., 2021; Yasué et al., 2019), changes are unlikely to persist and may lead to shallow or performative engagement and resentment that could ultimately thwart autonomous motivation and long‐term behavior change (Chamberlin et al., 2023; Legault et al., 2007; Vallerand et al., 1997). Simply put, autonomous motivation predicts long‐term behavior change, whereas controlled motivation does not (Deci et al., 2001; Ryan & Deci, 2017) (Figure 1).

A self‐determination theory lens is useful not only because both CBC and JEDII require sustained, long‐term interventions, but also because an enforcement‐based approach with penalties and fines is unlikely to work in both these contexts. In conservation, for example, there are not enough resources to guard vast tracts of forested land, patrol distant coastal communities for illegal practices, provide long‐term economic incentives for conservation, or punish local inhabitants. Furthermore, punishments can lead to backlash (Cetas & Yasué, 2017; Coelho‐Junior et al., 2022; Sumaila et al., 2006). Similarly, rather than enforcement, fostering autonomous motivation toward JEDII is critical because the university is a decentralized institution, where faculty, in particular, have tremendous autonomy and academic freedom, and the threat of anti‐EDI backlash among disgruntled faculty members is significant (Dobbin & Kalev, 2022; Pihie et al., 2011; Snodgrass & Shachar, 2008). Deans, department heads, and other leaders rarely implement top‐down punitive approaches and instead inspire and lead within a model of collegial governance (Bisbee, 2007; Dea, 2021). In such a system and culture, staff, faculty, and leaders in universities make daily decisions about how to run classes, who to hire, what items to prioritize in a budget, who to interview, what policies or practices to advance, and who to profile on a website, all with implications for JEDII (Stewart & Valian, 2022). In such a system, the key to embedding change is to support individuals at every level of the institution to become autonomously motivated to advocate and support change toward JEDII. This approach includes the support of senior leaders who have the capacity to change policies and practices that can make it easier for others to engage in JEDII. An autonomy‐supportive approach is critical in universities because motivation is shared and amplified among individuals in organizational settings. Pressured individuals pass the pressure onto others, and inspired individuals will inspire others (Reeve & Cheon, 2021; Zimmer‐Gembeck et al., 2015). Furthermore, a punitive and controlling approach may work if the changes required are narrow, performative gestures or follow a checklist (Daigle, 2019; Kutlaca & Radke, 2023). However, because CBC and JEDII require creative, deep, widespread, and enduring change, fostering autonomous motivation is critical to success (Crompton & Kasser, 2010).

Given its importance for sustained behavior change, researchers in self‐determination theory have identified the types of interpersonal, organizational, and societal contexts that foster autonomous motivation (Deci et al., 2017; Martela et al., 2023; Ryan & Deci, 2000). Specifically, support for 3 basic psychological needs facilitates autonomous motivation: autonomy (behaving in line with one's values), competence (feeling effective and achieving meaningful goals), and relatedness (feeling connected to others and a sense of belonging) (Figure 1). Supporting these needs by cultivating ownership, belonging, skill development, and connection (Ryan & Deci, 2000), and avoiding controlling tactics, such as rewards, punishments, and shame, is key to embedding enduring change. We drew on examples from CBC and used self‐determination theory as a theoretical framework to identify principles (Table 1) to create the context for autonomous motivation for JEDII.

**TABLE 1:** In community‐based conservation (CBC) projects and justice, equity, decolonization, indigenization, and inclusion (JEDII) initiatives in postsecondary education, examples of how to support autonomy by providing meaningful choice and different entry points to engagement (Recommendation 1).

Action	CBC	JEDII
Present conservation and JEDII as a solution to meaningful concerns or problems.	For fishing communities, present CBC as potential solutions for reduced fish catch or size.	Present JEDII to department heads as potential solutions to interpersonal conflicts, poor teaching evaluations, and student or faculty recruitment challenges.
Collaboratively set realistic priorities and design processes for action based on existing resources, skills, and knowledge.	Ask communities what species and areas they want to conserve instead of developing a blueprint for conservation action elsewhere and attempting to export it globally. It may be realistic to set a goal of increasing coral cover in a small community reserve, but it is not a reasonable goal to rebuild the populations of internationally migratory fish populations.	Ask departments what JEDII action is most important to their units in the short and long term. Ask and listen to units as they describe their JEDII priorities rather than assuming, dictating, or mandating certain actions. It may be realistic goal to improve conflict engagement skills of people in leadership roles so that they can effectively manage a microaggression situation between a student and faculty member. However, it is not reasonable to set a goal of having no microaggressions or abolishing White supremacy from an institution. Consider first tackling less complex tasks. One may be able to embed JEDII into a 3‐person faculty departmental colloquium committee first and then delve into the much more complex task of revamping the departmental faculty tenure process.
Collaboratively decide how to measure and define success and when there is enough planning and analysis.	Engage communities in setting goals and indicators for success of interventions. Remind communities about the range of choices and options available to them. Involve communities in participatory research methods. Check in with communities about how much planning and data collection is enough.	
Create space for expression of negative feelings.	Listen to challenges and costs that farmers perceive about new regulations or actions of environmental organizations.	Listen to negative feelings and discomforts. For example, about time costs of JEDII, being publicly shamed, or concerns about negative teaching evaluations.
Provide compelling and meaningful reasons to help make sense of initially unpopular changes.	Explain to communities the constraints to actions by conservation if certain policies prevent preferred action. Explain why those constraints are in place in accessible language relevant to the community's existing goals, identity, and values.	If additional time is required to implement inclusive hiring processes, take the time to discuss the importance of each of these additional steps in hiring.
Engage community leaders and support existing efforts.	Build relationships with existing conservation champion, elders, and leaders who can help inform conservation and JEDII organizations about the existing priorities and approaches to conservation and JEDII in their communities. Be careful not to subsume existing community initiatives and instead support and amplify effective initiatives.	

*Note*: “Communities” in the context of conservation and in JEDII may refer to affinity groups, departments, or administrative units that work together and share common interests and goals.

## SUPPORT AUTONOMY BY CREATING MEANINGFUL CHOICE AND DIFFERENT ENTRY POINTS TO JEDII ENGAGEMENT (RECOMMENDATION 1)

### Community‐based conservation

CBC is more likely to be successful and foster autonomous motivation if the psychological need of autonomy is supported by the project (Table 1). Meeting this need means that the CBC project is community endorsed, and there are meaningful choices in the design, development, and implementation of conservation projects (Akers & Yasué, 2019; Ban et al., 2017; Cetas & Yasué, 2017; Dawson et al., 2021). Autonomy‐supportive CBC projects include active and diverse community participation from the start. The community identifies the problem, sets meaningful goals and priorities, and identifies measures of success and when there is sufficient information to take action (Alcorn, 1993; Dale & Armitage, 2011; Koster et al., 2012; Reo & Whyte, 2012). The community chooses their entry point for conservation. Conversely, conservation interventions that were imposed on communities or failed to reach all relevant rightsholders and stakeholders in communities (Kleiber et al., 2015; Shepherd, 2004; Youdelis, 2016) not only breach human rights but may also fail to conserve natural resources (Adams et al., 2004). A common type of autonomy‐thwarting CBC project is a conservation interventions devised in urban offices of development or conservation organizations and then exported into rural communities. These interventions fail to acknowledge the unique historical, sociocultural, and environmental contexts of the communities as well as, in some cases, existing long‐term governance and stewardship systems (Ostrom, 1990; Reyes‐García & Benyei, 2019). Without opportunities for substantive input and delegation of power (Arnstein, 1969), the CBC project seems irrelevant, distant, or infantilizing to communities (Brosius et al., 2005; Dowie, 2011). If there is little agreement on the goals and priorities for conservation, for example, even enthusiastic supporters of conservation may lose autonomous motivation and disengage or thwart conservation efforts (Alcorn, 1993; Bowen‐Jones & Entwistle, 2002; Yasué et al., 2019).

### Justice, equity, decolonization, Indigenization, and inclusion

Much like the work of autonomy‐supportive conservation outreach staff (Yasué et al., 2019, 2022), supporting the psychological need for autonomy requires an understanding of the values and challenges that specific units or roles in an institution face and the implementation of this knowledge to create meaningful entry points for JEDII action (Table 1). The goal is not to change values—values are relatively resilient to change over long periods (Crompton & Kasser, 2010; Manfredo et al., 2017)—but instead to support a sense of autonomy while doing JEDII work so that intrinsic values (those conducive to supporting JEDII) are supported (Sheldon et al., 2011). A skilled leader or staff member can support autonomy and meaningful choices by helping units relate JEDII to pressing priorities, identify different possible JEDII options, and implement achievable goals within the culture, knowledge, and capacity of a team or unit. For example (Table 1), units may identify changes in practices that are easier to implement for newer EDI committees (e.g., diversifying colloquium speakers or providing conflict engagement training in faculty retreats) versus more challenging and complex changes (e.g., changing faculty performance review processes or changing first year mandatory curricula). Through collaborative dialogue, units can develop meaningful, relevant, and timely actions for change.

One key challenge to creating significant organizational changes (such as the cultural transformation to embed JEDII into a university) in a manner that supports autonomy is the increasingly short tenures of senior leadership. Unlike the long‐term residents of a university community (i.e., long‐term faculty and staff), senior leaders move among universities to advance their careers (Monks, 2012, p. 20; Röbken, 2007). As JEDII becomes a priority for some institutions, new leaders are enticed to create new JEDII policies, programs, or initiatives to leave their mark on an institution. However, similar to the challenges of (Peckham et al., 2017; Yasué & Kirkpatrick, 2020) distantly devised conservation projects, without sufficient time to understand the experiences and cultures and codevelop interventions, these initiatives may thwart autonomy and fail to receive buy‐in and legitimacy.

## PRIORITIZE RELATIONSHIPS (RECOMMENDATION 2)

### Community‐based conservation

A multigenerational Tasmanian farmer said, “To be able to influence a mindset of a community is a great way of influencing change… Communities are strong. Relationships are strong. Human expectations of each other are higher than any other motivation” (Yasué et al., 2019). Strong, reciprocal relationships (Pound, 2011; Trosper, 2009) built on trust and created over time (Baker, 2017; Jenkins et al., 2017; Koster et al., 2012) are central to supporting the need for relatedness and creating resilient sociocultural change (Table 2). Strengthening relationships within communities and between communities and external agencies can help foster better decision‐making, efficient allocation of resources, and sustainable and resilient initiatives (Jenkins et al., 2017; Ostrom, 2009; Pretty & Smith, 2004). People are inspired to act by seeing key respected peers, colleagues, and leaders create change (Cialdini et al., 2006; Nichols & Safina, 2004; Yasué et al., 2022). These examples suggest that conservation initiatives were successful when needs‐supportive staff took the time to listen deeply, celebrate successes, and listen to descriptions of challenges that affect the lives of these communities (Peckham et al., 2017; Yasué & Kirkpatrick, 2020). Relationships rooted in reciprocity, friendship, respect, and empathy provide the foundation for partnerships and sociocultural change (Nichols & Safina, 2004; Oldekop et al., 2016).

**TABLE 2:** In community‐based conservation (CBC) projects and justice, equity, decolonization, indigenization, and inclusion (JEDII) initiatives in postsecondary education, examples of how to support the need for relatedness through prioritizing relationships (Recommendation 2).

Actions	CBC	JEDII
Learn about the culture, values, and histories of other groups.	Ask farmers about their personal stewardship values and interests. If it is aesthetics, describe how planting trees can improve aesthetics. If it is concern for future generations, connect trees to conservation water reservoirs for future generations.	Invest time in learning about the ceremonies and cultures of historically marginalized groups, for example, by reading books, attending events, or listening to music. Ask faculty how JEDII might support a unit's strategic goals and aspirations. If goals are about research excellence, provide information on how diverse and interdisciplinary teams conduct more impactful research.
Normalize and value relationship building as part of professional skills.	Ensure that project proposals engage in long‐term relationship building prior to the initiation of a project.	Support competence by teaching leaders how to run meetings or facilitate workshops that allow people to learn about and care for each other. Include relationship building with diverse groups as part of teaching, research, and service responsibilities in performance review of faculty. If there is a group of leaders who want to increase the number of Indigenous students, have the leaders go to a remote community and learn and meet the family and students they are trying to recruit.
Create colearning, connection, and care opportunities for communities from different groups.	Build a network of fishing communities concerned about poaching. Create social and learning events that bring poachers and conservationists together to create change.	Create affinity groups and communities of practices of JEDII champions to provide mentorship and role models. Hire warm and empathic JEDII advisors to coach, consult, and support.
Lead with empathy and perspective‐taking during conflict.	When people poach, attempt to understand the perspectives and challenges that led to poaching. Cocreate conservation projects that help reduce some of the root causes of poaching (e.g., poverty or insufficient access to protein).	Build competence by training faculty to respond thoughtfully and listen carefully when members of marginalized groups express experiences related to marginalization. Ensure that people feel valued and a sense of belonging even when their views or behaviors are questioned, challenged, or prohibited (e.g., when harmful to others).
Enhance relationship building skills.	Proactively teach people conflict engagement skills to help people maintain relations despite conflicting perspectives. Advance and support the career trajectory of skilled relationship‐builders. Hire and recruit leaders who are skilled in maintaining relationships through differences and embody the values of creating an inclusive community and one in which people feel valued even as their attitudes or behaviors are challenged. Hire warm, empathic, autonomy‐supportive environmental stewardship officers and JEDII advisors to build relationships with different people and help repair relationships after conflict.	

*Note*: Communities in the context of conservation and in JEDII may refer to affinity groups, departments, or administrative units that work together and share common interests and goals.

### Justice, equity, decolonization, indigenization, and inclusion

Given the power of departmental relationships in supporting or thwarting organizational change, cultivating relationships with and between the long‐term residents of this community is critical to building autonomous motivation and creating change to support JEDII (Festré & Garrouste, 2015; Lingras et al., 2023). The deep‐seated and cultural changes necessary for people to feel autonomously motivated to engage in JEDII are more likely to happen through multiple conversations with people they trust and share common interests (Balietti et al., 2021). Less relational approaches, such as reading a new scientific paper, attending a 15‐min didactic mandatory training on bias (Carter et al., 2020; Mobley & Payne, 1992), or receiving incentives for attending workshops, are not as likely to foster a sense of connection, belonging, and understanding.

Universities need to prioritize and fund relationship building as a key step to meeting strategic priorities related to JEDII (Table 2). Relationship building could include creating like‐minded groups, such as JEDII communities of practice (e.g., JEDII champions in leadership roles) or affinity groups (e.g., Black, Indigenous, People of Color, women in science) (McCoy & Bocala, 2022). These groups not only spur autonomous motivation and resilience to engage in JEDII but also support well‐being and retention especially of marginalized groups (Myers et al., 2019; Pour‐Khorshid, 2018; Tatum, 2017). It is not sufficient for university leaders to merely indicate that such relationships and affinity groups are important and to occasionally offer affinity spaces immediately after a heated moment when racism is salient (e.g., anti‐Asian hate; the murder of George Floyd; Palestine, Israel, Gaza conflict). It is critical that they proactively provide funds for ongoing relationship building. Leadership support is important for affinity groups because of the logistical challenges of building community across large organizations and because the decision to support affinity spaces (which are by definition closed off to certain groups of people) may receive backlash, despite having demonstrated benefits for marginalized groups (Hughes et al., 2022; Ohito & Brown, 2021; Pour‐Khorshid, 2018).

In addition to developing affinity spaces (Jenkins et al., 2017), it is also critical to build strong connections between different groups. These connections could include bridges between different departments that have implemented JEDII, between units and central EDI units, and between units advocating for Indigenization and decolonization as well as inclusion, equity, and accessibility. These bridges support resource sharing and deepen empathy and understanding across actors on the challenges and opportunities to engage in JEDII from different positions.

Prioritizing relationship building also means hiring and training leaders who are empathic, relational, and effective at intentionally building community and developing initiatives across diverse groups (Ginwright, 2022) and restoring relationships and promoting growth after conflict (Llewellyn et al., 2015; Mehl‐Madrona & Mainguy, 2014; Miller‐Jones & Rubin, 2021). Valuing and recruiting for these types of skills might require asking questions about relationship building or conflict engagement in interviews or discussing relationship building in annual performance review processes (Ramsey & Latting, 2005).

## DESIGN FUNDING PROGRAMS AND OTHER EXTRINSIC INCENTIVES THAT ENHANCE AUTONOMOUS MOTIVATION (RECOMMENDATION 3)

### Community‐based conservation

A multigenerational Tasmanian farmer said, “…payments [for conservation] get people doing something but if it is not in the spirit, you are not agreeing on the outcome that you want. It is just somebody …doing it because somebody is paying them to do it. You aren't going to get any change in belief or change in behavior, which ultimately will undo whatever it is that you are doing” (Yasué et al., 2019). Funding is critical for implementing CBC (Ferraro & Pattanayak, 2006; Milne & Niesten, 2009). How these resources are provided can have positive or negative impacts on autonomous motivation. If funds are perceived as rewards or handouts designed to control behavior (Deci et al., 2001), this conditional reward can negatively affect relationships and crowd‐out autonomous motivation (Akers & Yasué, 2019; Yasué et al., 2019). However, if funds cultivate a sense of partnership and competence (because it allows people to make the changes they wish to make), this method can support all basic psychological needs and increase autonomous motivation (Deci et al., 2001; Ezzine‐de‐Blas et al., 2019). For Tasmanian farmers (Yasué et al., 2019), stewardship payments from government to farmers supported autonomy and relatedness by building collaborations and allowed farmers to share the risk of taking on new environmental management schemes. When speaking about these payments, one multigenerational Tasmanian farmer said, “The payments have never met our economic needs, it's been very symbolic …but its symbolic nature is such that it's enormous in terms of you feeling that someone else is coming [on] the journey with you.”

### Justice, equity, decolonization, indigenization, and inclusion

Creating a context within JEDII to support autonomy is not the same as neglect. Financial resources are critical to foster a sense of competence and build relationships to create change (Clark et al., 2023) (Table 3). For JEDII work, resources are particularly important to mitigate the minority tax (Rodríguez et al., 2015) in which marginalized groups disproportionately engage in JEDII work without being adequately compensated. Although marginalized groups may apparently autonomously volunteer, it may be due to fear that others in dominant groups do not value the work and lack skills to engage in JEDII. Thus, feeling forced or have one's autonomy thwarted when taking on these obligations can negatively affect feelings of autonomy, belonging, and relatedness (Rodríguez et al., 2015; Trejo, 2020).

**TABLE 3:** In community‐based conservation (CBC) projects and justice, equity, decolonization, indigenization, and inclusion (JEDII) initiatives in postsecondary education, examples of how to design payment programs and extrinsic motivators that support the need for autonomy (Recommendation 3).

Actions	CBC	JEDII
Provide funds to build and deepen relationships and design grants carefully to not “crowd‐out” internal motivation.	Consider framing payments for farmers to plant trees as a way of collaborating, building partnerships, and sharing costs rather than as an incentive or a reward. Consider providing grants to entire communities to build a guard house for a reserve rather than awarding payments to a single individual.	Create an equitable and transparent process for applying for JEDII grants. Develop relationships with fund recipients in order to provide ongoing support for units leading JEDII projects.
Avoid framing training as a means to avoid punishments and instead frame as meaningful learning.	Avoid threatening arrest as a justification for attending training and instead invite attendance by piquing interest, curiosity, and a desire to contribute to the community.	Avoid framing JEDII training and education as a way of avoiding problems from professional associations, getting fired, being part of a costly human rights complaint, losing research grants, or avoiding critical social media posts from students. Instead frame learning as an opportunity to grow and innovate and be a better doctor, engineer, lawyer, or educator.
Avoid implementing harsh punishments and instead apply autonomy‐supportive techniques to support learning and relationship building.	Don't kill or imprison people who have illegally hunted or socially ostracize people who have engaged in illegal fishing methods. Instead, reframe such infractions as an opportunity to learn about the root causes (e.g., poverty, disengagement from the reserve) and meet gaps in knowledge and understanding in an autonomy‐supportive manner.	As a unit head, do not publicly shame or dismiss people who have said the wrong word or made a JEDII mistake and instead highlight mistakes as a way to support learning and relationship building. Avoid mandating training as a punishment. Instead, allow mistakes to create opportunities for empathy, deep listening, connection, and collaborative action to create long‐term and proactive systems change.
Implement autonomy‐supportive language and approaches.	Avoid use of controlling language, such as *should*, *must*, and instead invite people to reflect, choose, and consider full range of possible options available to them. “Here are a range of options that you might consider to move JEDII forward, which of these resonate the most for you?” When hiring leaders, value leaders who are skilled in incorporating autonomy‐supportive language and who inspire a team rather than relying on extrinsic motivators to create change.	
Develop funds to equitably, and transparently fund work.	Provide resources so busy leaders and community members are able to execute plans (e.g., provide funds for administrative support, lunches to build relationships). Equitably share the workload and create plans for some ongoing support because both CBC and JEDII work take time. Ask communities how they want to be compensated for their CBC and JEDII work (e.g., individual payments, a new school, course release).	

*Note*: Communities in the context of conservation and in JEDII may refer to affinity groups, departments, or administrative units that work together and share common interests and goals.

Creating transparent, equitable, accessible, and low‐barrier sources of funding can expand the range of possible actions (thus support autonomy and competence), strengthen relationships by helping to spread the reputational or economic risks, and expand existing community‐endorsed promising initiatives (Kamceva et al., 2022; Stein, 2020). These funds can stimulate work on the iterative process of creating changes in policies and practices to support JEDII. Funding could be administered through a transparently evaluated, equitable grant process in which units apply to support projects that are meaningful for the unique cultures of their unit and work toward institutional JEDII commitments. These funds could be supported by psychological‐needs‐supportive staff who build relationships with units, provide feedback on proposals, collaborate in problem‐solving, and support units in creating meaningful JEDII change. By fostering personal development, enabling action, supporting community building of actors involved in funded projects, and helping to enhance the salience of intrinsic values, these autonomy‐supportive staff ensure the funds support cultural change. It is critical to explicitly channel these funds to build stronger relationships between all 3 groups: the centralized JEDII unit, the administrative unit providing the funds, and the unit receiving the funds. Fund recipients should also build relationships with each other. For example, similar to learning exchanges in CBC in which farmers visit each other's farms (Yasué et al., 2020, 2022), grant recipients can connect with each other and inspire future recipients through similar exchanges. Such efforts not only build the descriptive norm that enables change (Cialdini et al., 2006) but also allow people who are not JEDII experts to support their autonomous motivation by sharing ideas, inspiration, and enthusiasm (Dedeurwaerdere et al., 2016; McCoy & Bocala, 2022; Suarez‐Balcazar et al., 2023).

Similar to the research on motivational crowding out in conservation (Akers & Yasué, 2019), in addition to providing resources in ways that do not crowd out autonomous motivation to engage in JEDII, it is critical not to frame JEDII with autonomy‐thwarting extrinsic language and motivators, such as fines, legal penalties, punishments, jeopardizing accreditation, or mandatory training (Dobbin & Kalev, 2022; Mobley & Payne, 1992; Sanchez & Medkik, 2004). Instead of providing extrinsic motivators, one should frame JEDII in terms of values that many academics endorse, such as personal growth, learning, innovation, and inclusive excellence (Stewart & Valian, 2022).

## DEVELOP MEANINGFUL EDUCATIONAL OPPORTUNITIES THAT ARE RELEVANT, TIMELY, RELATIONAL, AND AUTHENTIC (RECOMMENDATION 4)

### Community‐based conservation

Educational approaches may be more likely to promote autonomously motivated changes in behavior if the education is meaningful and relevant and rooted in cocreation, partnership, and trust (Rajecki, 1982; Shanley, 2006; Trewhella et al., 2005). In contrast, conservation scholars once believed that educating homeowners, farmers, and poachers about threats to resource scarcity, instilling fear of punishment or environmental impacts, would lead people to change attitudes and subsequently change behaviors, such as harming endangered species (de Weerd & Degens, 2019; Kollmuss & Agyeman, 2002; Saberwal, 1997). However, 1980s behavioral science led to a clearer understanding that the didactic approach of filling deficits of information may not shift behaviors and long‐term commitment to conservation (Ardoin et al., 2020; DeCaro & Stokes, 2008; Wells & Lekies, 2006). Educational approaches that support deeply held values related to fairness, inclusion, and prosociality are more likely to succeed than approaches that foster shame, compliance, or fear of punishment (Crompton & Kasser, 2010; Sheldon et al., 2011).

### Justice, equity, decolonization, indigenization, and inclusion

For JEDII, much can be learned from incorrect assumptions made about conservation education decades ago. The most common tool to create the deep, transformative change that is needed to shift attitudes, values, practices, and policies toward JEDII has become the didactic short antiracism or implicit bias training from an external JEDII expert (Carter et al., 2020; Dobbin & Kalev, 2018). A cynical view is that these training sessions are an attempt to check off JEDII without actually forcing institutions to change the systems by integrating JEDII into policies or procedures. The presence of JEDII workshops is not the end‐goal of JEDII, and indeed having these workshops can create the false impression that real transformative change is happening, when it is not (Dobbin & Kalev, 2018). Although education can play an important role in creating change, the currently prevalent one‐off awareness sessions may not support autonomous motivation to engage in JEDII, may fail to have long‐lasting behavioral impacts (Carter et al., 2020; Chang et al., 2019; Goulden et al., 2019; Legate & Weinstein, 2024), and can backfire (Daumeyer et al., 2019; Legault et al., 2011). Below, we offer suggestions for creating more effective and lasting JEDII learning opportunities (Table 4).

**TABLE 4:** In community‐based conservation (CBC) projects and justice, equity, decolonization, indigenization, and inclusion (JEDII) initiatives in postsecondary education, examples of how to develop meaningful educational opportunities so individuals can benefit from relevant, timely, relational, and authentic education that supports the needs of autonomy, competence, and relatedness (Recommendation 4).

Actions	CBC	JEDII
Provide relevant information to aid in decision‐making.	Provide communities with fish transect survey results, maps of coral reef cover, or biological information about species of interest to aid communities in decision‐making.	Provide JEDII leaders in units with lists of possible courses of actions and a range of short how‐to tip sheets to help consider actions. Inspire by providing units with examples of different JEDII action that is happening in other units.
Cocreate meaningful, timely, and relevant tailored educational engagement for a diversity of audiences.	Ongoing and relevant training throughout the lifetime of a CBC project. For marine reserves, this might include awareness building at the start and moving on to more action‐oriented training, such as managing budgets in later years (Yasué et al., 2022).	Create different types of trainings on JEDII for different groups (e.g., leaders, administrative assistants, faculty, dominant groups, marginalized groups) in different formats to help different roles embed JEDII into their work. For time‐pressed leaders, one‐page tip sheets on specific types of integration of JEDII into roles and responsibilities might help people who care about JEDII but don't know how to effectively integrate them into practices (e.g., tip sheet on JEDII and interview techniques, or JEDII in research).
Meet with units to consult and discuss rather than dictate learning.	Have staff visit farms to understand the context where people work to provide suggestions on how communities might integrate conservation into their work. Hire outreach staff who can provide rich informational feedback to farmers and can draw on personal farming experience to provide meaningful and relevant advice.	Have autonomy‐supportive staff meet communities and provide information‐rich feedback, encouragement, and meaningful scaffolding on conservation or JEDII projects that are meaningful to units. Avoid mandatory, time‐consuming, and cookie‐cutter education that may seem disconnected from problems they face in their jobs and instead inspire people to attend sessions that use realistic case scenarios and are relevant.
Create opportunities to interact and reflect in workshops.	Provide opportunities for people to connect JEDII and conservation to their existing roles and responsibilities. Create opportunities for people to build community and interact with each other to discuss relevant tricky scenarios.	
Resource long‐term competence‐supportive staff.	Work collaboratively to help prioritize or break down complex and challenging tasks (e.g., decolonizing higher education, resilience to climate change) into a set of smaller achievable tasks for each year—for example, improving job descriptions to broaden the skills we seek in faculty, creating a welcoming website, or planting a set of trees and changing the types of vegetation to grow surrounding a field.	

*Note*: Communities in the context of conservation and in JEDII may refer to affinity groups, departments, or administrative units that work together and share common interests and goals.

Efforts toward JEDII should be codesigned and cofacilitated with someone inside the organization. Partnering with a unit leader to create a workshop can develop meaningful educational opportunities with relevant scenarios, examples, and historical contexts (Shanley, 2006). A unit leader can ensure that the topics covered connect JEDII to the department or discipline's institutional commitments or strategic priorities (which participants are more likely to already endorse). Collaborations support psychological needs by not only deepening the leader's understanding and capacity to facilitate these types of workshops on their own but also helping to strengthen relationships between units and EDI advisors. In contrast, inviting external JEDII experts who have limited local context or relationships may fail to engage people who are not already on board with JEDII and could strengthen the incorrect notion that external experts are needed because academics do not know enough about this topic and therefore cannot implement change. This tactic can end up delaying action even though most fields and people have some relevant lived and professional experience related to JEDII.

#### Create JEDII workshops that empower rather than shame participants

Often trainings provide definitions about microaggressions, White supremacy, privilege, and bias without helping people connect JEDII to their work (Chang et al., 2019; Dobbin & Kalev, 2018). Such workshops may thwart competence and instill fear of making mistakes. Focusing on shame or blame can lead to defensiveness, strengthen controlled motivation (rather than autonomous motivation) (Iyer, 2022; Phillips & Lowery, 2015), and lead to refusal to engage in conversations and change toward JEDII. Instead of focusing on the nuances of continually changing language and identification of all the ways in which mistakes can be made, training needs to build relevant skills and provide time for people to connect JEDII to their day‐to‐day activities so that people feel a sense of competence and are empowered to embed JEDII into their roles to create change (Cocchiara et al., 2010; Dobbin & Kalev, 2018). For example, workshops can provide intergroup or cross‐cultural dialogic skills so that leaders can facilitate respectful, productive, and meaningful communications for long‐term, sustainable, and iterative interpersonal learning. In addition, workshops can provide scripts on recovering from mistakes, repairing relationships, and cultivating a growth mindset that views mistakes as opportunities for learning or growth or chairing inclusive meetings. These skills can promote empathy, cultural humility, and perspective‐taking within groups and help groups learn from the diverse experiences, perspectives, cultures, social identities, functions, and disciplinary fields within a group. Creating practical skills for effective conversations on heated, potentially polarizing, and emotional topics, such as EDI and identity, is critical to realizing change within units (Balietti et al., 2021; Maxwell et al., 2012). Providing time in workshops for busy leaders to reflect and plan allows leaders to consider different perspectives (Welker et al., 2023) and strengthen the connection between their own responsibilities and JEDII. These opportunities help build empathy toward other groups, build a sense of competence to effect change, and remind participants that they have choice (autonomy) and power to create change (Lindsey et al., 2015).

#### Create interactive opportunities during workshops

Work in JEDII is not solitary. It is collaborative and requires skills in collective decision‐making. This interactive approach is missing in some workshops in which the majority of the time may be more of a passive lecture model of education (King, 1993). Instead, with parallels to the effectiveness of active learning in classrooms (Freeman et al., 2014) and students as cocreators, where relationship building is central (Bovill, 2020), nonexpert people should have opportunities to show what they know, build a sense of community, connect readings and ideas to knowledge they already hold, and share the challenges they face and solutions they attempted (Bruin et al., 2018; Roberson et al., 2001). Group problem‐solving and collaboration with realistic scenarios and situations can nurture curiosity and inner motivations through inspiration and joint problem‐solving in addition to deepening learning and promoting autonomy (Koh et al., 2008; Wood, 2003).

In addition to creating opportunities for people in the same unit to interact and discuss JEDII, it is important to provide focused educational opportunities for people with similar roles across units (Bruin et al., 2018; Roberson et al., 2001). These groups can share successes, exchange ideas between units, promote a positive descriptive norm in a trusted peer (Balietti et al., 2021), and build a sense of competence and belonging so that autonomous motivation is retained.

#### Consult, advise, and coach to create change

Other types of learning opportunities may be more effective than one‐off group workshops. Authentic education (Lee et al., 2022) through confidential small‐group and one‐on‐one coaching, consultation, motivational interviewing, just‐in‐time learning, and mentorship in real‐life contexts (Friedman et al., 2021; Gabriel et al., 2014; Hettema et al., 2005; Mehra & Inman, 1992; Sarid, 2015; Verplanken & Orbell, 2022) can change habits (or existing practices) and build motivation to create deep, transformative changes in policies, practices, and culture. Academics are generally curious and intrinsically motivated to engage in complicated problems and puzzles. Active opportunities to work through real projects could tap into creativity, deep engagement and a “flow state” (Nakamura & Csikszentmihalyi, 2014) that build people's interest in learning and growing. With autonomy‐supportive advisors or peers who can support champions and leaders coupled with budgets and informational resources, the task of changing practices and policies may become optimally challenging, engaging, and invigorating.

## CREATE INSTITUTIONAL CHANGE BY FOCUSING EFFORTS ON CRITICAL MOMENTS (RECOMMENDATION 5)

### Community‐based conservation

One of the major challenges in CBC and JEDII is to engage more people in action (Nadkarni, 2004; Yasué & Kirkpatrick, 2020). Relative to this challenge, an idea from conservation literature is that of hot moments for change. Taken from the concept of biodiversity hotpots (i.e., key locations to conserve) (Myers et al., 2000), hot moments are moments of critical importance to conservation (Radeloff et al., 2013), for example, moments of social change (e.g., independence from colonial rule, new municipal council, or new owner of a multigenerational farm [Table 5]) or upheaval (e.g., conflicts).

**TABLE 5:** In community‐based conservation (CBC) projects and justice, equity, decolonization, indigenization, and inclusion (JEDII) initiatives in postsecondary education, examples of how to support autonomy, relatedness, and competence by creating institutional change through focusing efforts on critical moments (Recommendation 5).

Actions	CBC	JEDII
Embed learning and institutional change after a conflict.	Restore relationships between poachers and conservationists to promote colearning and target the root causes of poaching.	Avoid naming, shaming, punishing, or shunning when mistakes or conflicts occur. Build restorative processes in institutions. Build training programs to create a culture of openness, empathy, and perspective taking and support conflict literacy among leaders. Identify procedural changes that were the cause of conflict and develop a plan to address these changes after a conflict in order to create long‐lasting change.
Support learning for new leaders and practitioners.	Build relationships with new local government officials, landowners, department heads, or deans. When hiring leaders, include interview questions that relate to growth and learning after a conflict to ensure that leaders have the skills to work through heated moments in a productive manner. Embed conservation or JEDII into existing professional development programs and fishing or farming educational programs to help embed conservation in agricultural or fishing practices.	

*Note*: Communities in the context of conservation and in JEDII may refer to affinity groups, departments, or administrative units that work together and share common interests and goals.

High‐conflict moments with increased attention and resources can provide opportunities for collaborative planning among traditionally polarized groups. In the 1970s, a highly publicized 13‐year conflict between the Haida Nation of Haida Gwaii in northwest British Columbia and the logging industry led to the landmark Gwaii Hanaas Agreement in which the Haida Nation and the federal government agreed to comanage the Gwaii Haanas National Park Reserve, National Marine Conservation Area Reserve, and Haida Heritage Sites as equal parties (Hawkes, 1996; Jones et al., 2017; Wheatley, 2006). This agreement, which remains one of the most significant examples of comanagement globally, occurred not only because of the resilience and leadership of the Haida Nation but also because environmental organizations, forestry companies, as well as local and federal governments overcame differences to identify common goals.

### Justice, equity, decolonization, indigenization, and inclusion

There are also hot moments for change in JEDII (Table 5). If JEDII practitioners have close relationships to various departments, they can detect hot moments when there is a higher chance of creating change. One hot moment for change is the onboarding of new leaders and faculty. These people are at the start of potentially long careers at the same institution, arrive with ideas from the outside, and are at a formative point in their lives. This fresh start is a hot moment in their careers, at a time when they are setting habits and learning a new environment. Timely experiential learning for this population, linked to mentorship and developing skills in the primary aspects of their jobs (e.g., teaching and research), can help set and evolve their long‐term personal norms and expectations. For example, paired teaching, in which an experienced faculty member and an incoming faculty member coteach a well‐designed, student‐focused course, can have lasting impacts on a faculty member's future inclusive teaching practices (Holland et al., 2018; Moghtader et al., 2022). Over time, with the cycle of hiring and retirements, faculty who have had this initial formative experience dominate the unit and contribute to cultural change.

Similar to the case of Gwaii Haanas Agreements, moments of escalating societal conflict, particularly those attracting media attention, often raise awareness, interest, and pressure for resolution from a broader group of rightsholders and stakeholders. For example, the murder of George Floyd by police in the United States led to the global Black Lives Matter protests (Applewhite, 2021). In Canada, decades of advocacy for Indigenous rights went largely unseen until May 2021 when 215 unmarked graves of children were found at the former Kamloops Indian Residential School in British Columbia, spurring protests across Canada as people sought greater accountability and action toward truth and reconciliation (Gulliver, 2021; Truth and Reconciliation Commission of Canada, 2015). Both of these events led to a sudden flurry of interest and media attention (Barrie, 2020). For many Black and Indigenous people, this media attention brought to the surface the systemic and interpersonal racism they experienced (Eichstaedt et al., 2021). However, for many people, especially those with the privilege of not previously thinking about racism, these events were the first time they learned about widespread intergenerational and systemic racism.

Beyond the common practice of reactive, empty, performative statements of support and remorse (Daigle, 2019), it is critical that universities capitalize on these catalytic events that provide moments of heightened relevance to create proactive systemic change. The urgency stemming from these events, within broader shifts in public opinion about ongoing injustice and inequities, often leads to increased time and financial resources supporting JEDII (Llewellyn et al., 2015). In these moments, these topics seem more relevant to the community and allow busy leaders and faculty to prioritize action and learn more about these issues. This timing is particularly important for people from dominant groups who are less engaged in JEDII (Bruin et al., 2018). These moments create a meaningful on‐ramp to engage new people. With more people, especially people with formal power engaged, there is greater potential for novel collaborations and relationship building between groups with different skills and roles and formal and informal power that is necessary to action structural, systemic, and transformative change (Applewhite, 2021).

Increasingly, there are university conflicts related to JEDII (Gronert, 2019; Llewellyn et al., 2015; Tate, 2022). In these moments of conflict, extrinsic pressures due to fears about litigation and reputation of the unit or individuals may push leaders toward inaction, silence, and attempts to control or suppress the conflict by applying top‐down authority. However, as the costs and visibility of conflict mount, frustrated and polarized faculty (Ahern, 2018) protected by academic freedom turn to social media to express frustrations or confusion, and avoidance becomes untenable (Ahmed, 2021). Rather than framing discussions in these contexts as mere compromises, leaders could seize the opportunity to resolve these issues in a way that builds intergroup engagement skills and creates systemic change (Fisher & Ury, 1981). Once the need to break entrenchment has been identified by the key parties, de‐escalation efforts, provided there is careful support, can foster more autonomous motivations and maintain a growth mindset (Dweck, 2006) within groups to address conflict. By practicing deep empathic listening and creative collaborative problem‐solving, these moments can create new communities, rebuild existing ones, foster learning, create change, and build more long‐term and proactive solutions. In conservation case studies, moments when poachers become heroes (Nichols & Safina, 2004; Peckham et al., 2017) are critical to create changes in self‐perception and identity and develop empathy, autonomous motivation toward JEDII, and common humanity across a group.

## CONCLUSION

As people begin to engage in JEDII work, it can feel overwhelming because they realize the myriad changes needed to shift an institution. Much like conservation, there are competing priorities and complexities, gaps in knowledge, many papers describing the way things can go wrong, critical media coverage on actions (or inaction), and little guidance on the singular best way. However, it is critical to act. In addition to reputational risks and reduced institutional legitimacy, not acting, especially for people with marginalized identities in conservation and in academia, can lead to gas lighting, despair, hopelessness, increasing polarization, reduced well‐being, and reduced commitment and engagement in conservation or JEDII in universities (Ahern, 2018; Trejo, 2020).

Shifting entrenched academic institutions and norms is hard and will take time. Conditional rewards and punishments can create short‐term, superficial behavioral changes, but creating social contexts to support autonomous motivation is critical to tackling the numerous actions and structural changes for the long and ever‐improving journey of JEDII work. It is a group marathon, not an individual sprint. It requires deep buy‐in and continual commitment and proactive and timely reactive action. In this context, these motivational principles and their subsequent strategies for action can be implemented across the university and within CBC to produce behavioral and systemic change.

We demonstrate how CBC experiences and self‐determination theory can identify principles to create change toward more effective and enduring interventions. Specifically, we demonstrate how satisfying needs for autonomy, competence, and relatedness to foster autonomous motivation can identify concrete practices to create meaningful choice, build reciprocal relationships, resource JEDII, and create capacity building and educational opportunities to integrate JEDII into the work of universities. Self‐determination theory may not be a silver bullet for all JEDII or CBC challenges, and there are perhaps moments when extrinsic approaches are necessary; however, the extensive research on the outcomes of autonomous motivation leads us to advocate for a more autonomy‐supportive approach.

Change cannot happen through the overworked and emotionally and economically taxed members of historically marginalized groups (Rodríguez et al., 2015) in only the small number of fields that are directly connected to social justice and equity (e.g., Indigenous Studies, Gender Studies). Instead, it is critical to engage people with dominant identities in different types of roles and disciplines in universities (Kutlaca & Radke, 2023; Mitchell & Bishop, 2020). This engagement includes conservation scholars. A critical first step to shifting away from small performative action and the minority tax toward the goal of deep structural change and fostering autonomous motivation for more diverse groups to engage is for all scholars to carefully consider the relationship between their own academic fields, identities, or roles and JEDII. Here, we started the process to strengthen the relevance between the field of conservation and JEDII by exploring the unique histories and approaches in CBC and identifying important entry points, insights, and strengths for conservation scholars in terms of implementing JEDII. This type of analysis is critical to help build the meaningful buy‐in necessary for a team of autonomously motivated, empowered, creative, and diverse JEDII supporters to create relational, interdisciplinary, and enduring systemic change.
